# Neuroprotective effect of *Angelica gigas* root in a mouse model of ischemic brain injury through MAPK signaling pathway regulation

**DOI:** 10.1186/s13020-020-00383-1

**Published:** 2020-09-22

**Authors:** Se-Eun Lee, Jung-Hoon Kim, Chiyeon Lim, Suin Cho

**Affiliations:** 1 Department of Korean Medicine, School of Korean Medicine, Yangsan Campus of Pusan National University, Yangsan, 50612 Republic of Korea; 2 Department of Medicine, College of Medicine, Dongguk University, Goyang, 10326 Republic of Korea

**Keywords:** *Angelica gigas* (apiaceae), Ischemic stroke, Infarction, Neuroprotection, Pharmacokinetics

## Abstract

**Background:**

The root of *Angelica gigas* Nakai (Apiaceae) has been traditionally used as an important herbal medicine to treat blood-deficiency-related disorders in Eastern Asian countries, and recently, it has been recognized as a potential candidate for improving cardiovascular diseases.

**Methods:**

In this study, the neuroprotective effect of a methanol extract of *A. gigas* root (RAGE) was investigated in a mouse stroke model induced by a 90 min transient middle cerebral artery occlusion (tMCAO). Infarction volumes and morphological changes in brain tissues were measured using TTC, cresyl violet, and H&E staining. The neuroprotective mechanism of RAGE was elucidated through investigation of protein expression levels using western blotting, IHC, and ELISA assays. The plasma concentrations of decursin, a major compound in RAGE, were measured after oral administration of RAGE to SD rats.

**Results:**

The infarction volumes in brain tissues were significantly reduced and the morphological deteriorations in the brain neuron cells were improved in tMCAO mice when pre-treated with RAGE at 1000 mg/(kg bw·d) for two consecutive days. The neuroprotective mechanism of RAGE was confirmed to attenuate ERK-related MAPK signaling pathways in the ipsilateral hippocampus hemisphere in mice. The concentrations of decursin in rat plasma samples showed peak absorption and elimination in vivo after oral administration of RAGE at 100 mg/rat.

**Conclusion:**

Mice administered RAGE before the tMCAO operation had less neuronal cell death than those that were not administered RAGE prior to the operation, and this study provides preclinical evidence for use of *A. gigas* in ischemic stroke. 
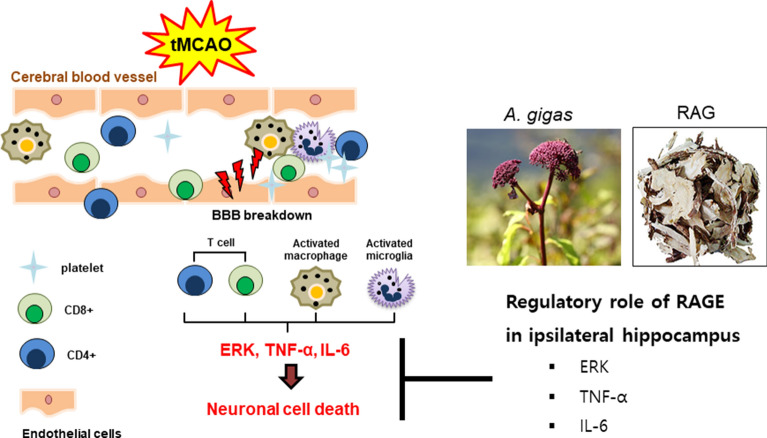

## Background

Acute ischemic cerebral stroke is considered the leading cause of morbidity and mortality in modern society [1–5]. When ischemic stroke occurs, cerebral inflammation and cell death are induced in the ischemic lesions, and inflammatory signals are activated by harmful stimuli such as arterial occlusion [6–9].

Ischemic stroke causes high morbidity and disability, with many patients needing rehabilitation and long-term care resulting in a massive financial burden on healthcare systems [9, 10]. Thrombolytic agents, such as anticoagulant or antiplatelet agents, have been used for the treatment of stroke with some success. However, there are limitations to their use, including side effects such as hemorrhage, and the limited window of opportunity for efficacious administration. Thus, further research on the prevention and treatment of ischemic stroke is essential [11, 12].

*Angelica gigas* Nakai (Apiaceae), also called Korean angelica, is a perennial plant from Asian countries, and in traditional herbal medicine, its roots are used to treat symptoms or disorders caused by blood-deficiency [13]. Several studies have investigated the chemical components and pharmacological activities of *A. gigas* [13–21]. In Korea, the root of *A. gigas* (RAG) has traditionally been used as a representative blood circulation agent when derived from either source [13, 14].

According to traditional Korean medicine, strokes are mainly caused by blood stasis, and RAG has been widely used by traditional Korean medicine practitioners to treat ischemia-related diseases [13–15]. Pharmacologically, it has been reported to have anticancer and antibacterial effects, antioxidant activity, and to improve circulatory diseases activity [15–19]. More recently, research has indicated that its components have neuroprotective and anti-inflammatory effects [20, 21].

Therefore, in this study, we investigated the mechanism of neuroprotective action of the methanolic extract of the root of *A. gigas* (RAGE) on a mouse model of ischemic stroke induced by a 90 min transient middle cerebral artery occlusion (tMCAO). Moreover, pharmacokinetic parameters of decursin, a dominant compound in *A. gigas* root, were determined by comparing plasma samples from rats orally administered RAGE at a dose corresponding to that administered to tMCAO mice.

## Materials and methods

### Chemicals and reagents

Analytical methanol, acetonitrile, and water were purchased from J.T. Baker Inc. (Phillipsburg, NJ, USA). LC/MS grade acetonitrile and water (containing 0.1% formic acid) were purchased from Fisher Scientific (Pittsburgh, PA, USA). Ethyl acetate was obtained from SK Chemicals (Seongnam, Gyeonggi-do, Korea). Trifluoroacetic acid was purchased from Sigma-Aldrich (St Louis, MO, USA). Nodakenin, decursin, and schizandrin (internal standards, IS) were purchased from the Korea Food and Drug Administration (Osong, Chungbuk, Korea). Chlorogenic acid and imperatorin were purchased from ChemFace (Wuhan, Hubei, China). All marker compounds had ≥ 98% purity.

PBS was purchased from Bio Basic Inc. (Markham, Ontario, Canada). 2,3,5-Triphenyl-tetrazolium chloride (TTC) and cresyl violet were purchased from Sigma-Aldrich (St. Louis, MO, USA). Saline was obtained from JW Pharmaceutical Co., Ltd. (Seoul, Korea). The optimal cutting temperature compound for cryostat embedding medium was purchased from Thermo Fisher Scientific (Waltham, MA, USA). Methanol was purchased from SK chemicals (Ulsan, Korea). Protein extraction solution was purchased from iNtRON (Seongnam-si, Gyeonggi-do, Korea). Primary antibodies for *p*-ERK (#9101), ERK (#9102), *p*-JNK (#9255), JNK (#9252), *p*-p38 (#9211), and p38 (#9212) were purchased from Cell Signaling Technology (Danvers, MA, USA). Goat polyclonal secondary anti-rabbit (ADI-SAB-301-J) and anti-mouse (ADI-SAB-101-J) IgG antibodies were obtained from Enzo Life Sciences Inc. (Farmingdale, NY, USA). West-Q chemiluminescent substrate was purchased from GenDEPOT (Katy, TX, USA). BCA reagent, BSA standard, and enhanced chemiluminescence western blotting chemiluminescent substrate were purchased from Thermo Fisher Scientific (Waltham, MA, USA). Enzyme-linked immunosorbent assay (ELISA) kits for TNF-α and IL-6 was purchased from Abcam (Cambridge, UK).

### Preparation of RAGE

The dried root of *A. gigas* was purchased from the Kwangmyungdang Medical Herbs Co. (Namgu, Ulsan, Korea). RAG (200 g) was extracted with 99% methanol at 25℃ for two days to obtain the methanolic extract. The extract was filtered and concentrated again using a rotary vacuum evaporator (EYELA, Tokyo, Japan), and then, the concentrate obtained (34 g) was designated RAGE.

### Preparation of RAGE for high performance liquid chromatography (HPLC) analysis and analytical conditions

RAGE (10 mg) was dissolved in methanol (1 mL) and filtered through 0.2 μm syringe filters (BioFact, Daejeon, Korea). The filtrate was prepared for HPLC analysis at a final concentration of 1000 μg/mL by dilution with methanol.

HPLC analysis of four marker compounds was performed using an Agilent 1200 liquid chromatography system (Agilent Technologies, Palo Alto, CA, USA) equipped with an autosampler, degasser, quaternary solvent pump, and diode array detector. Separation of the marker compounds was performed on a Capcell Pak Mg II C_18_ column (4.6 mm × 250 mm, 5 μm; Shiseido, Tokyo, Japan) at 35℃ using a flow rate and an injection volume set at 1 mL/min and 10 μL, respectively. The mobile phase consisted of water containing 0.1% trifluoroacetic acid (solvent A) and acetonitrile (solvent B) and the gradient elution was as follows: 10% (B) for 0–5 min, 10–20% (B) for 5–10 min, 20% (B) for 10–33 min, 20–50% (B) for 33–35 min, 50% (B) for 35–60 min, 50–90% (B) for 60–61 min, 90% (B) for 61–65 min, and then, re-equilibrated to 10% (B) until 70 min. The diode-array detector was set at ultraviolet wavelengths of 250 nm (for imperatorin), 325 nm (for chlorogenic acid), 330 nm (for decursin), and 335 nm (for nodakenin).

### Experimental animals

Wild-type male C57BL/6 mice and SD rats were supplied by Samtako (Incheon, Korea). Animals were housed at constant temperature (22 ± 2℃) and relative humidity (50 ± 10%) conditions under a 12 h light/dark cycle for 7 days with free access to feed and water before the experiments. Animals were fasted overnight before surgery or oral administration of RAGE. Animal experiments were conducted according to the guidelines for animal experimentation issued by the Pusan National University and the experimental procedures were approved beforehand by the Animal Ethics Committee of Pusan National University (PNU–2018–2113 and PNU–2019–2124).

### Induction of tMCAO

Mice (aged 7 to 9 weeks, weighing 21 to 25 g) were exposed to 90 min tMCAO with an intraluminal filament. Mice were anesthetized using 1.5–2% isoflurane in N_2_O/O_2_ (70%/30%). In anesthetized mice, the left side of the common carotid artery was exposed and isolated. The middle cerebral artery (MCA) was occluded by inserting an 8–0 surgical coated-monofilament nylon suture into the internal carotid artery, which was advanced further until it closed the origin of the MCA. During the experiments, MCA blood flow was monitored using a laser Doppler flowmeter (MoorVMS-LDF, Moor Instruments Ltd., UK) connected to a single fiber optic probe adhered onto the skull surface of the core area supplied by the left. The reduction and maintenance of relative cerebral blood flow (rCBF) under 20% of pre-ischemic baseline was used to confirm the occlusion, and rCBF recovery more than 40% of the pre-ischemic baseline was considered as successful reperfusion. Rectal temperature was maintained at 37 ± 0.5℃ by means of a heating blanket and heating lamp throughout the surgery. Sham-operated control mice received the same surgical procedure without insertion of a filament.

### TTC staining

Mice were quickly anesthetized with isoflurane, and the brains were removed rapidly and placed in ice-cold water for 3 min. Coronal slices of 1 mm thickness were prepared, and sections were immersed in 2% TTC at 37℃ for 17 min. The presence of infarction was determined by the area that was stained negative with TTC.

### Neurological deficit scores (NDS) assessment

NDS assessments were performed by investigators who were blinded to the experimental groups, as described previously [22]. The following rating scale was used: 0 = no deficit, 1 = failure to extend right forepaw, 2 = decreased grip strength of right forepaw, 3 = circling to right by pulling the tail, and 4 = spontaneous circling.

### Western blot analysis

The ipsilateral cortex and hippocampus of tMCAO mice were homogenized in PRO-PREP protein extraction solution. After centrifugation, the supernatants were used for immunoblot analysis. *p*-ERK, *p*-JNK, JNK, *p*-p38, p38 (diluted 1:2000), and ERK (diluted 1:1000) were used as the primary antibodies. Immunodetection was performed and quantified using a photosensitive luminescent analyzer system (Amersham™ Imager 600, MA, USA) according to the protocol provided by the manufacturer. All bands were analyzed using ImageJ (NIH, MD, USA).

### Cresyl violet, hematoxylin and eosin (H&E), and immunohistochemistry (IHC) staining

For cresyl violet staining, sections were incubated at 40℃ for 10 min. Slides were placed directly in chloroform:alcohol overnight. Then, the sections were stained with cresyl violet at 40℃ for 40 min. After washing under water for 10 s, sections were quickly dehydrated in a 95% alcohol series, and placed in xylene for 1 min. The slides were sealed with a cover slip and mounting solution. Differences between the treatment groups were compared under an optical microscope.

For H&E staining, sections were incubated at 40℃ for 10 min. Then, slides were passed through 85% alcohol for rehydration, and washed under water for 2 min. The sections were stained with hematoxylin for 10 min and then washed under water for 1 min to remove the excess dye from the tissue. The sections were stained with eosin Y solution for 1 min. After washing under running water for 1 min, air-dried sections were passed through 85% and 99% alcohol series and dewaxed in xylene for 1 min. The slides were sealed with a cover slip and mounting solution. Differences between the treatment groups were compared under an optical microscope.

For IHC staining, primary antibodies for TNF-α and IL-6 were used at a 1:200 dilution. The sections were incubated overnight at -4℃ in primary antibodies. The sections were washed, and incubated with secondary antibody for 1 h each at room temperature. IHC analysis was performed using the DAKO Envision kit (DAKO, Carpinteria, CA, USA).

### ELISA assay

To detect TNF-α or IL-6 in ipsilateral hippocampus proteins and blood serum, we used an ELISA kit. The samples and standard samples were diluted with distilled water and applied to ELISA plates. The TNF-α or IL-6 concentrations were determined according to the manufacturer’s instructions. Absorbance levels were measured at 450 nm using an ELISA reader.

### LC/MS conditions for the pharmacokinetics study

Quantification of decursin was performed using an Accela LC system (Thermo Fisher Scientific; MA, USA) equipped with an autosampler, degasser, and quaternary solvent pump. Decursin and IS were separated on a Hypersil GOLD C_18_ column (2.1 mm × 100 mm, 1.9 μm; Thermo Fisher Scientific, MA, USA) at 35℃. The flow rate was set at 300 μL/min with an injection volume of 5 μL. The mobile phase consisted of water (containing 0.1% formic acid, A) and acetonitrile (B) and the gradient elution applied was as follows: 30–60% (B) for 0–4 min, 60% (B) for 0.5 min, and then re-equilibrated to 30% (B) until the end of the analysis.

An LCQ Fleet ion-trap mass spectrometer (Thermo Fisher Scientific; MA, USA) was used to detect compounds in the eluent using the electrospray ionization source in the positive-ion mode. MS conditions were as follows: sheath gas (nitrogen), 50 arbitrary units; auxiliary gas (nitrogen), 20 arbitrary units; spray voltage, 5.0 kV; capillary temperature, 300℃; and capillary voltage, 30.0 V. Quantification of the compound was performed in the selective ion monitoring mode at 329 m*/z* [M+H]^+^ for decursin and 415 m*/z* [M+H]^+^ for the IS. Data was processed using Excalibur (v. 2.1.0; Thermo Fisher Scientific, CA, USA).

### Preparation of stock solutions, calibration standards, and plasma sample

Accurately weighed decursin and IS were dissolved in methanol at a concentration of 100 μg/mL, and the solution was diluted with methanol to prepare working solutions for the construction of the calibration curve. Calibration standards were prepared using 150 μL of blank rat plasma spiked with 10 μL of working solutions and 10 μL of IS solution, which was extracted using ethyl acetate.

Plasma samples were prepared using a liquid–liquid extraction method. An aliquot of 150 μL plasma sample was spiked with 10 μL of IS solution and 10 μL of methanol, and 800 μL of ethyl acetate was added. Extraction of decursin and IS was performed by vortex mixing for 2 min at 25℃, followed by centrifugation at 704 × *g* for 10 min at 20℃. The upper layer (ethyl acetate) was then transferred to a clean 1.5 mL polypropylene tube and evaporated using a nitrogen gas blowing concentrator (MGS–2200; EYELA, Miyagi, Japan). The residue obtained after evaporation was reconstituted in 100 μL of methanol, vortex mixed for 2 min, and centrifuged at 704 × *g* for 10 min at 20℃. The supernatant was transferred to a glass vial and a 5 μL aliquot was injected into the UPLC–MS/MS system.

### Pharmacokinetics study

RAGE (100 mg) was dissolved in ethanol solution (ethanol:saline = 1:9, *v/v*) and was orally administered to SD rats (*n* = 6). Blood samples (-0.3 mL) were collected from a jugular vein in heparinized tubes before the administration, and 0.17, 0.5, 1, 2, 4, 6, 8, 12, 24, or 48 h after intragastric administration. Collected blood samples were immediately centrifuged at 704 × *g* for 10 min, and 150 μL aliquots of supernatant plasma were transferred to clean tubes and stored at -20 °C until analysis. The pharmacokinetic parameters of decursin were calculated using plasma concentration of decursin versus time data using PKSolver (a free add-in program for Microsoft Excel) [23].

## Statistical analysis

Data are presented as the mean ± standard error and analyzed using SigmaPlot 12.0 (Systat, CA, USA). Statistical differences between groups were evaluated using ANOVA followed by the Dunnett’s test, and values of *p* < 0.05 were considered significant.

## Results

### HPLC analysis of RAGE and its standard compounds

**Fig. 1:**
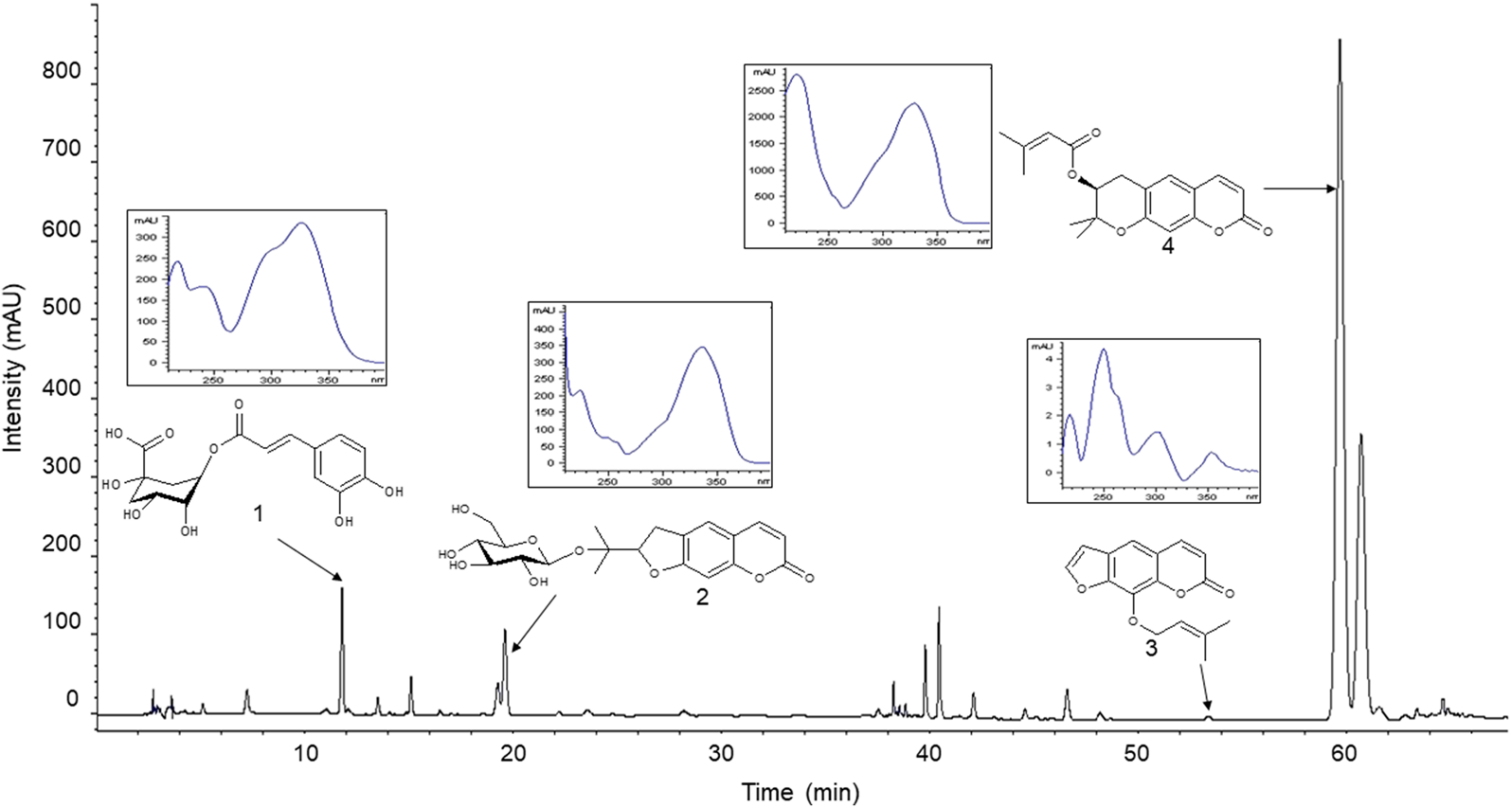
Chromatograms of RAGE at the UV wavelength of 250 nm with chemical structures and UV spectra of the marker compounds: 1. chlorogenic acid, 2. nodakenin, 3. imperatorin, and 4. decursin

Four marker compounds were confirmed in RAGE by their retention times and UV spectra compared with those of the standard compounds on the HPLC chromatogram (Fig. 1) and their content in the extract was as follows: 11.303 ± 0.188 mg/g for chlorogenic acid, 25.923 ± 0.241 mg/g for nodakenin, 0.191 ± 0.005 mg/g for imperatorin, and 141.130 ± 1.309 mg/g for decursin (Table 1).

**Table 1:** Qualitative identification and quantitative calibration of four marker compounds in RAGE

Compound	λ_max_ (nm)	t_*R*_ (min)	Calibration equation	*r*^2^	Calibration range (μg/mL)
Chlorogenic acid (1)	218, 326	11.76	y = 23.414x—4.5529	0.9991	0.78–50.00
Nodakenin (2)	224, 336	19.58	y = 23.670x + 8.2782	1.0000	3.13–200.00
Imperatorin (3)	218,250,302,354	53.33	y = 52.339x + 0.5308	0.9998	0.31–10.00
Decursin (4)	222,326	59.67	y = 56.013x + 82.431	1.0000	9.38–600.00

### Effects of RAGE on infarct volumes and NDS

**Fig. 2:**
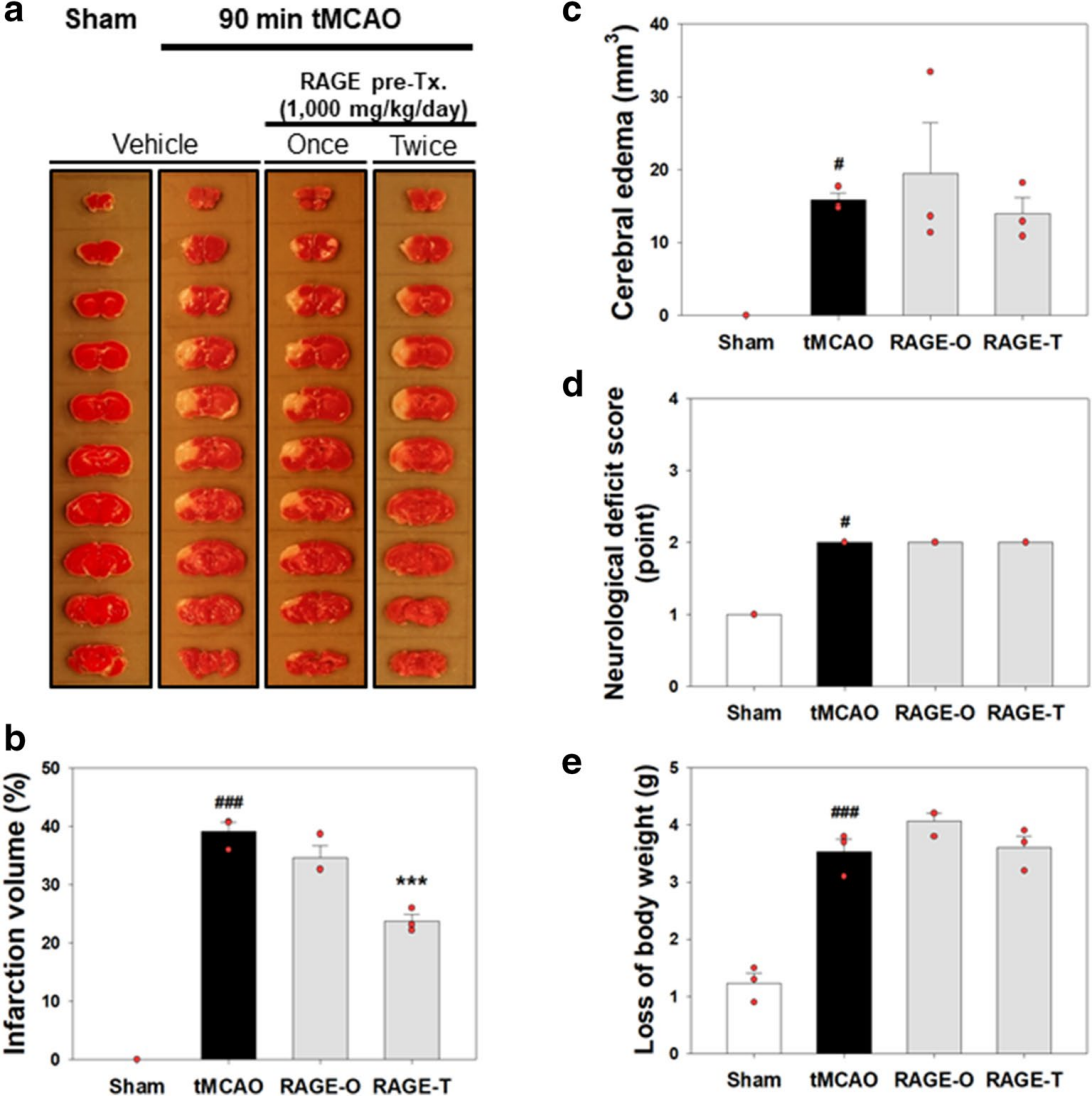
Measurements of infarction volume, edema area, NDS, and body weight change.** a** representative photographs of TTC-stained brain slices (1 mm) showing the infarct area 24 h after tMCAO treatment;** b** quantitative analysis of the total infarct volume;** c** quantitative analysis of the total edema area;** d** NDS;** e** change in body weight. Once, single RAGE treatment; twice, RAGE treatment for two consecutive days at 1000 mg/(kg bw·d) RAGE. All data are expressed as mean ± standard error (n = 3). ^#^ *p* < 0.05, ^###^ *p* < 0.001 vs. sham-operated group; *** *p* < 0.001 vs. tMCAO group

To study the effect of RAGE, mice were pre-treated with RAGE before induction of ischemic stroke by 90 min tMCAO. RAGE (1000 mg/kg bw) was administered once (1 h prior to tMCAO) or twice (1 h prior to tMCAO and 24 h earlier). The more effective regimen was the double 1000 mg/(kg bw·d) dose (Fig. 2a, b). The total infarct volume percentile in the ipsilateral brain hemisphere in mice treated twice with RAGE at 1000 mg/(kg bw·d) was 23.78 ± 1.14%, and that of the untreated tMCAO group was 39.16 ± 1.58% (Fig. 2b).

No significant reduction in the amount of brain edema was observed in the groups pre-treated with 1000 mg/(kg bw·d) RAGE once (19.48 ± 7.01 mm^3^) or twice (14.01 ± 2.19 mm^3^), when compared with the amount of edema seen in the untreated tMCAO group (15.88 ± 0.93 mm^3^) (Fig. 2c). No significant changes were observed in the neurological deficit scores (NDS) and body weight changes between the tMCAO-induced groups (Fig. 2d, e, respectively).

### Effects of RAGE on protein expressions

**Fig. 3:**
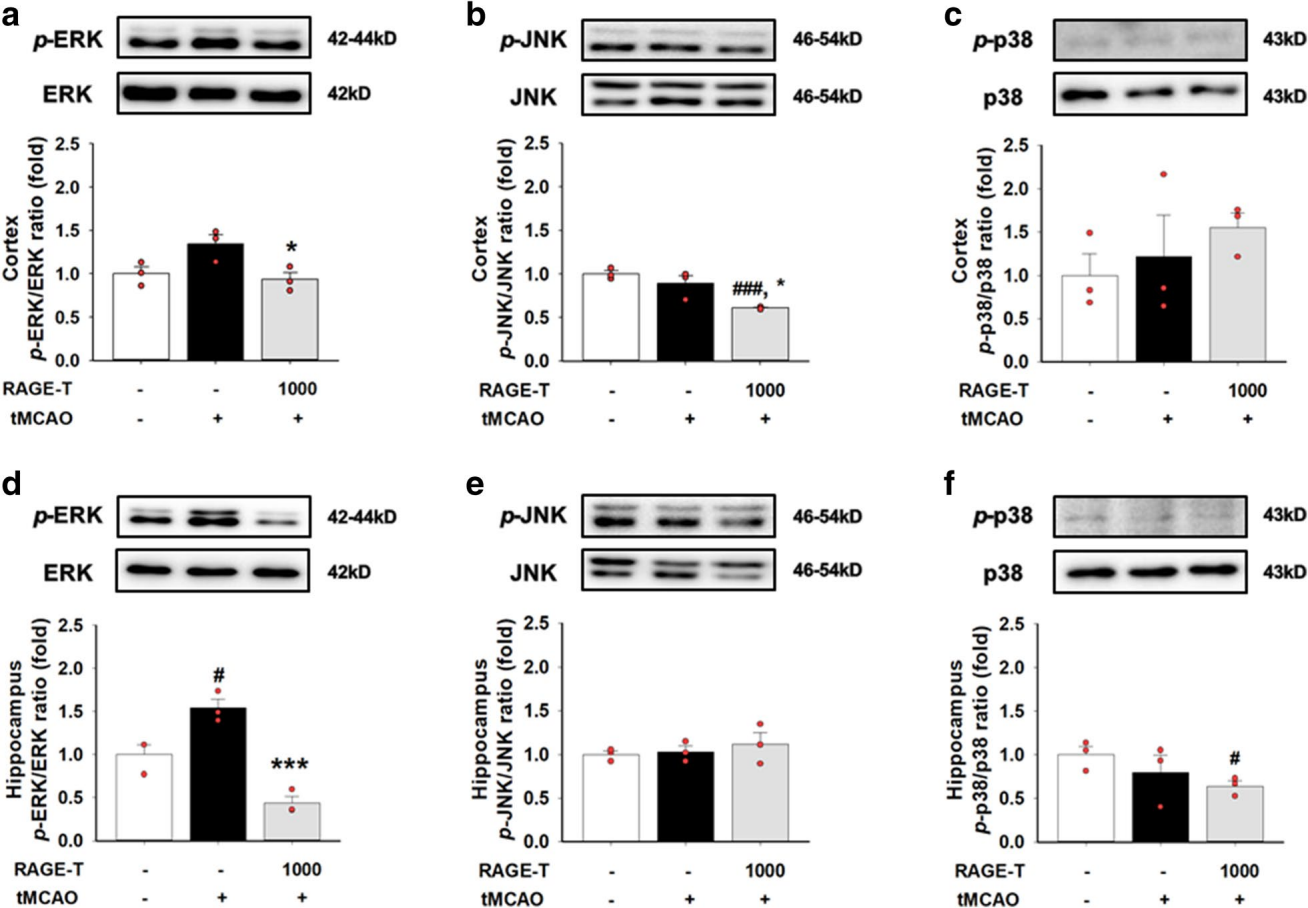
Effects of RAGE pre-treatment on protein expression in the tMCAO-induced mouse brain.** a**–**c**, representative image of western blot analysis of the expression of p-ERK, ERK, p-JNK, JNK, p-p38, p38, and β-actin in the ischemic ipsilateral cortex of the brain.** d**–**f**, representative image of western blot analysis of the expression of p-ERK, ERK, p-JNK, JNK, p-p38, p38, and β-actin in the ischemic ipsilateral hippocampus of the brain. All data are expressed as mean ± standard error (n = 3). ^#^*p* < 0.05, ^###^*p* < 0.001 vs. sham-operated group; * *p* < 0.05, *** *p* < 0.001 vs. tMCAO group

As pre-treatment of 1000 mg/kg bw RAGE twice was found to be more effective than the single treatment, we assessed the expression of cellular stress-related molecules in the ischemic ipsilateral cerebral cortex and hippocampus. tMCAO showed no significant changes of cellular stress-related protein expressions such as extracellular-signal-regulated kinase (ERK), c-Jun N-terminal kinase (JNK), and p38 mitogen-activated protein kinase (p38) (Fig. 3a–c); but interestingly, pre-treatment with RAGE increased phosphorylation of JNK (Fig. 3b). Hippocampal protein phosphorylation of ERK was increased by tMCAO which was regulated by pre-treatment of 1000 mg/(kg bw·d) RAGE twice (Fig. 3d). ERK, JNK, and p38 mediate mitogen-activated protein kinase (MAPK) pathways, and MAPK is an intracellular signal-mediating molecule involved in various cell activities including cell proliferation, differentiation, survival, death, and deformation [24–28]. Thus, MAPK signaling was partially activated by ERK in the tMCAO group, and the activated signaling pathways were thought to be regulated by RAGE treatment (Fig. 3d).

### Effects of RAGE on histological changes during ischemic brain tissue injury

**Fig. 4:**
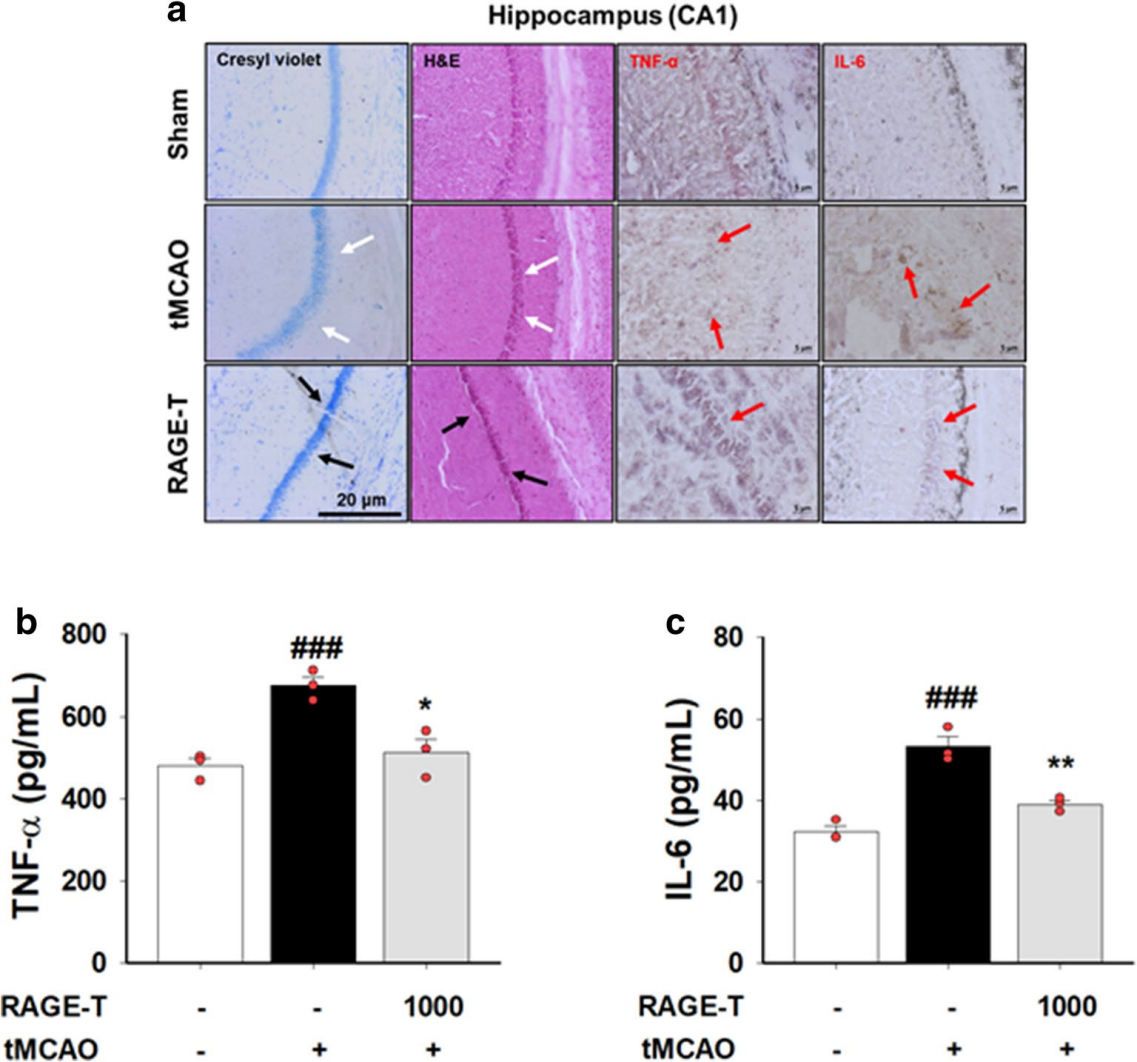
Neuroprotective effects of RAGE on tMCAO-induced hippocampal CA1 cell death. A, representative photomicrographs of cresyl violet-stained, H&E-stained, and IHC-stained (TNF-α and IL-6, respectively) hippocampal CA1 region. B and C, quantitative analysis of the TNF-α and IL-6 expression in tMCAO-induced hippocampal CA1 region using the ELISA method. All data are expressed as mean ± standard error (n = 3). ^###^ *p* < 0.001 vs. sham-operated group; * *p* < 0.05, ** *p* < 0.01 vs. tMCAO group

To evaluate the neuroprotective effect of RAGE on ischemic neuronal damage, we investigated the morphological changes to neuronal cells in the ischemic ipsilateral hemisphere of tMCAO-induced mice. In the sham-operated group, cresyl violet staining, also known as Nissl staining, indicated that the neuronal cells were intact, with a morphologically well-arranged cytoplasm and nucleus; however, in the tMCAO group, the neuronal cells in the hippocampal CA1 region were apoptotic, showing aberrant morphology with sparsely positioned cells (white arrows, Fig. 4a). In the RAGE-treated group, the cells were similar to those of the sham-operated group (black arrows, Fig. 4a). Hematoxylin and eosin (H&E) staining indicated that cell density of the hippocampal CA1 region in the tMCAO-induced group was slightly decreased, whereas in the group pre-treated with RAGE for two consecutive days cell density was similar to that of the sham-operated group (Fig. 4a). Hippocampal CA2 and CA3 regions showed a similar result as that of the CA1 region (Additional file 1: Figure S1).

## Effects of RAGE on pro-inflammatory cytokine levels

Immunohistochemistry (IHC) staining in the ischemic ipsilateral hemisphere showed that TNF-α and interleukin 6 (IL-6) were highly expressed in the hippocampal CA1 region (red arrows indicate TNF-α and IL-6 positive stains, Fig. 4a). The expression levels of TNF-α and IL-6 were lower in the RAGE-treated group than in the tMCAO group.

The effects of pre-treatment with RAGE twice on hippocampal inflammation after tMCAO-mediated injury were evaluated by measuring the levels of pro-inflammatory cytokines, TNF-α and IL-6. The ischemic ipsilateral hippocampus hemisphere of mice with tMCAO exhibited higher concentrations of TNF-α (675.87 ± 20.86 pg/mL) and IL-6 (53.25 ± 2.44 pg/mL) than that in the sham-operated mice (TNF-α; 480.73 ± 18.33 pg/mL, IL-6; 32.35 ± 1.44 pg/mL). However, RAGE pre-treatment significantly suppressed the expression of TNF-α (512.86 ± 32.96 pg/mL) and IL-6 (39.07 ± 0.97 pg/mL) (Fig. 4b, c). The serum levels of TNF-α and IL-6 showed no change with tMCAO-mediated brain injury (Additional file 1: Figure S2a, b); however, RAGE pre-treatment for two consecutive days significantly increased serum level of IL-6 (Additional file 1: Figure S2a, b).

### Pharmacokinetic change of decursin, the major compound of RAGE

**Fig. 5:**
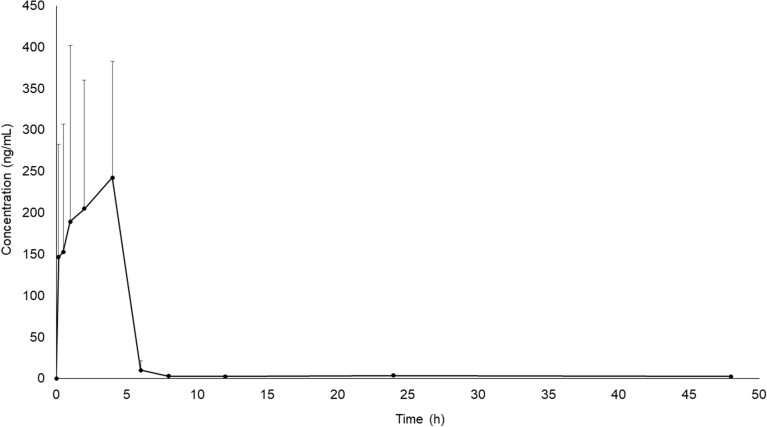
Chemical structures of decursin and schizandrin (IS), and mean plasma concentration–time curves of decursin after the oral administration of RAGE. Results are presented as mean ± standard (n = 6)

Decursin content in the plasma samples was determined after a single administration of RAGE to SD rats (100 mg/rat), a dose corresponding to that administered to tMCAO mice. The mean plasma concentration–time profile of decursin is shown in Fig. 5. The pharmacokinetic parameters, including area under the plasma concentration–time curve, maximum plasma concentration, time to reach the maximum plasma concentration, *t*_1/2_, mean residence time, and last measured concentration are shown in Table 2.

**Table 2:** Pharmacokinetic parameters of decursin after oral administration of RAGE

Parameter	Mean ± SD
AUC_0-∞_ (ng·hr/mL)	1218.319 ± 326.042
AUC_0-*t*_ (ng·hr/mL)	1186.935 ± 323.500
*C*_*max*_ (ng/mL)	407.888 ± 85.732
*T*_*max*_ (hr)	1.750 ± 1.255
*t*_*1/2*_ (hr)	8.283 ± 0.538
MRT_0-∞_ (hr)	6.897 ± 0.954
MRT_0-*t*_ (hr)	5.433 ± 0.581
C_last_ (ng/mL)	2.621 ± 0.665

AUC_0-*t*_, the area under the curve from 0 time to 48 h; AUC_0-∞_, the area under the curve from 0 time to infinity; *C*_max_, the maximum drug concentration in plasma; *t*_1/2_, half-life; *T*_max_, time taken to reach maximum drug concentration in plasma; MRT_0-∞_ (hr), mean residence time from 0 to infinity last measured concentration; MRT_0-__*t*_ (hr), mean residence time from 0 time to 48 h; C_last_, last measured concentration

## Discussion

Stroke is the second most common cause of death in the world. The most common type of stroke is ischemic stroke, a major neurological disorder that causes physical and psychological disorders [1–5]. As there is little regeneration of brain tissue after ischemic injury, there is a high possibility of a stroke relapse owing to cerebrovascular disorder. Therefore, drug therapy to improve ongoing risk factors is recommended for patients with post-ischemic brain dysfunction [29–31].

According to the theoretical basis of Korean medicine, pathological wind contains internal factors caused by liver dysfunction, fire is a stressful condition that can cause stroke, sputum can cause hyperlipemia or cerebral thrombosis, and a lack of life can cause a lack of blood circulation in the body [13, 32]. RAG has been widely used in various cardiovascular diseases as it has been reported to affect cardiovascular retarded vasodilation, neuroglutamic acid toxicity, and memory impairment [13]. Therefore, these various neuroprotective properties indicate that RAG could be used to treat ischemic stroke.

There have been previous reports regarding RAG and its constituents’ neuroprotective effects [13, 14, 25], and Shin et al. and Oh et al. reported that aqueous extracts of RAG attenuated cerebral damage and neuronal death in tMCAO-induced ischemic rats; however, they did not measured rCBF using laser Doppler flowmeter during the MCAO surgery [13, 33]. However, to our knowledge, pharmacokinetics studies on effective dosage of RAG have not been conducted until now. Furthermore, Shin et al. used rats as experimental animal, applied single treatment using intraperitoneal injection [13]. In our preliminary study, however, we used mice applying experimental methods of Shin et al., but we failed to confirm the effect of RAGE in that experimental condition. Therefore, we have newly identified conditions for the effect of RAGE in this study applying methanol as extraction solvent, and oral administration pathways. Thus, we demonstrated the pharmacological effects of RAGE in a previously reported modified ischemic stroke mouse model [22], and conducted a pharmacokinetic study with decursin, a major compound in the root of *A. gigas*.

RAGE reduced the total infarct volume induced by ischemic stroke when it was administered for two consecutive days prior to the ischemic stroke (Fig. 2A and 2B), and the *t*_1/2_ of decursin, a major bioactive compound of *A. gigas*, was 8.28 h after oral administration (Table 2), which suggests that the interval of administration should be strictly maintained to obtain high efficacy without adverse effects from RAGE. Although pre-treatment with RAGE once or twice showed no significant change in brain edema and NDS when compared to that in tMCAO group, administration of the treatment twice was considered to have higher efficacy than a single administration of the treatment. The dose of 1000 mg/(kg bw·d) of RAGE was tittered in our preliminary studies. A lower dose (under 300 mg/(kg bw·d)) showed no effects and a higher dose (over 3,000 mg/(kg bw·d)) showed adverse effects; therefore, we assumed that 1000 mg/(kg bw·d) would be more appropriate for this study. A RAGE dose of 300 mg/(kg bw·d) is closer to the clinically applied and recommended dose for daily use; however, in this rodent study, we assessed one or two administrations of 1000 mg/(kg bw·d) RAGE.

The effect of RAGE pre-treatment on tMCAO-induced cerebral damage was also assessed histologically in the hippocampal region. The cresyl violet stain for neurons and the H&E stain for nuclei and cytoplasm in the hippocampus CA1 region showed that pre-treatment with RAGE twice resulted in improved cell density and morphology when compared with that seen after treatment with tMCAO alone (Fig. 3a), and tMCAO-induced cerebral inflammation was inhibited by RAGE pre-treatment (Fig. 3sa-c). The cerebral inflammatory response induced by tMCAO did not seem to acutely affect parts of the body other than the brain (Additional file 1: Figure S2).

Corresponding results were obtained with western blotting in the ischemic ipsilateral hippocampus hemisphere showing inhibition of increased phosphorylation of ERK induced by tMCAO (Fig. 3d). MAPK is an intracellular signaling molecule with effects on cell migration, proliferation, and differentiation, and its relevant sub-molecules are ERK, JNK, and p38 [34–36], and activation of ERK promotes cellular growth, differentiation, or mitosis [26–28]. Ischemic ipsilateral hippocampal ERK signaling was significantly activated in the tMCAO group which was inhibited by RAGE pre-treatment (Fig. 3d). Interestingly, JNK signaling which activates apoptotic pathways by up-regulating pro-apoptotic genes [25–27] was down-regulated by RAGE pre-treatment (Fig. 3b).

Among the subgroups of the MAPK family, ERK is regarded as being related to cell survival; however, emerging evidence suggests the activation of ERK may lead to neuronal cell death, and inhibition of the ERK pathway is considered neuroprotective against oxidative stress [37]. Till date, the role of ERK remains controversial [34–37]. In our study, *p*-ERK was over-expressed in the MCAO-induced ipsilateral brain, and was down-regulated with the administration of RAGE (Fig. 3d). Although it is not yet possible to draw a definite conclusion, it may be assumed that ERK, a cell survival related signal, is activated by cell death related signals induced by MCAO, and was down-regulated by reduced brain injury owing to the administration of RAGE. Son et al. reported that activities of ERK and p38 in colon carcinoma cells were inhibited by decursin and decursinol which are the main components of *A. gigas* [38], thus supporting the role of RAGE in ERK-related molecular mechanism.

Plasma concentration of a bioactive compound after oral administration in vivo can provide important information in deciding the bioactive dose of herbal extract for rodents of a brain disease model. Decursin was found to be the most abundant in RAGE in our experiment, as previously reported [39], and thus, plasma concentrations of decursin were determined after oral administration of RAGE to SD rats. Although mice were used in the tMCAO experiment, we chose rats to quantify the plasma concentrations of decursin, as rats are more advantageous than mice for continuous collection of blood over a period of 24 h. The dose administered to tMCAO-induced mice was converted to 100 mg/rat using calculations described in Nair et al. [40]. Pharmacokinetic variables, such as area under the plasma concentration–time curve, time to reach the maximum plasma concentration, maximum plasma concentration, *t*_1/2_, and mean residence time, were calculated as physiological parameters after oral administration of RAGE to the non-diseased rat model. However, no studies have previously reported the pharmacokinetics of the compounds of *A. gigas* at their bioactive concentrations in animal models with a specific disease. Therefore, further studies on the pharmacokinetic changes in bioactive compounds of RAGE in diseased animal models are necessary for decisions on effective dosage.

## Conclusions

Mice administered RAGE at 1000 mg/(kg bw·d) for two consecutive days before the tMCAO operation had less neuronal cell death than those that were not administered RAGE prior to the operation. This protective effect involved MAPK-related cellular stress signals. Decursin was the most abundant compound in RAGE and its pharmacokinetic parameters were determined physiologically. This study provides preclinical evidence for the neuroprotective use of *A. gigas* in the treatment of ischemic stroke.

## Supplementary information

**Additional file 1.** Details regarding cell density in the hippocampal region and serum level changes in TNF-α and IL-6 with tMCAO-mediated brain injury and pre-treatment with RAGE are available in additional information.

## Data Availability

Please contact corresponding authors for data requests.

## Abbreviations

ANOVA: Analysis of variance
ERK: Extracellular-signal-regulated kinase
H&E: Hematoxylin and eosin
IgG: Immunoglobulin G
IHC: Immunohistochemistry
IL-6: Interleukin 6
JNK: C-Jun n-terminal kinase
MAPK: Mitogen-activated protein kinase
MCA: Middle cerebral artery
NDS: Neurological deficit scores
p38: P38 mitogen-activated protein kinase
RAG: Root of *Angelica gigas*
RAGE: Methanolic extract of the root of *A. gigas*
rCBF: Relative cerebral blood flow
*t*_1/2_: Half-life time
tMCAO: Transient middle cerebral artery occlusion
TTC: 2,3,5-Triphenyl-tetrazolium chloride
