# Supplementary material for: Neuroprotective effect of Angelica gigas root in a mouse model of ischemic brain injury through MAPK signaling pathway regulation
Source: Chin Med. 2020 Sep 22;15:101. doi: 10.1186/s13020-020-00383-1 (PMC7509924; doi:10.1186/s13020-020-00383-1)
Supplement: Supplementary file 1 — Additional file 1. Details regarding cell density in the hippocampal region and serum level changes in TNF-α and IL-6 with tMCAO-mediated brain injury and pre-treatment with RAGE are available in additional information. [file 13020_2020_383_MOESM1_ESM.docx]

**Supporting Information**

**Neuroprotective Effect of *Angelica gigas* root in a mouse model of ischemic brain injury through MAPK signaling pathway regulation**

Se-Eun Lee^1†^, Jung-Hoon Kim^1†^, Chiyeon Lim^2^, Suin Cho^1*^

^1^Department of Korean Medicine, School of Korean Medicine, Pusan National University, Yangsan 50612, Republic of Korea

^2^Department of Medicine, College of Medicine, Dongguk University, Goyang 10326, Republic of Korea

**^†^** These authors contributed equally to this study.

*Correspondence : Suin Cho, M.D., Ph.D., Professor, Department of Korean Medicine, School of Korean Medicine, Yangsan Campus of Pusan National University, Yangsan 50612, Republic of Korea

Phone: +82-51-510-8457, Fax: +82-51-510-8420

Email: sicho@pusan.ac.kr


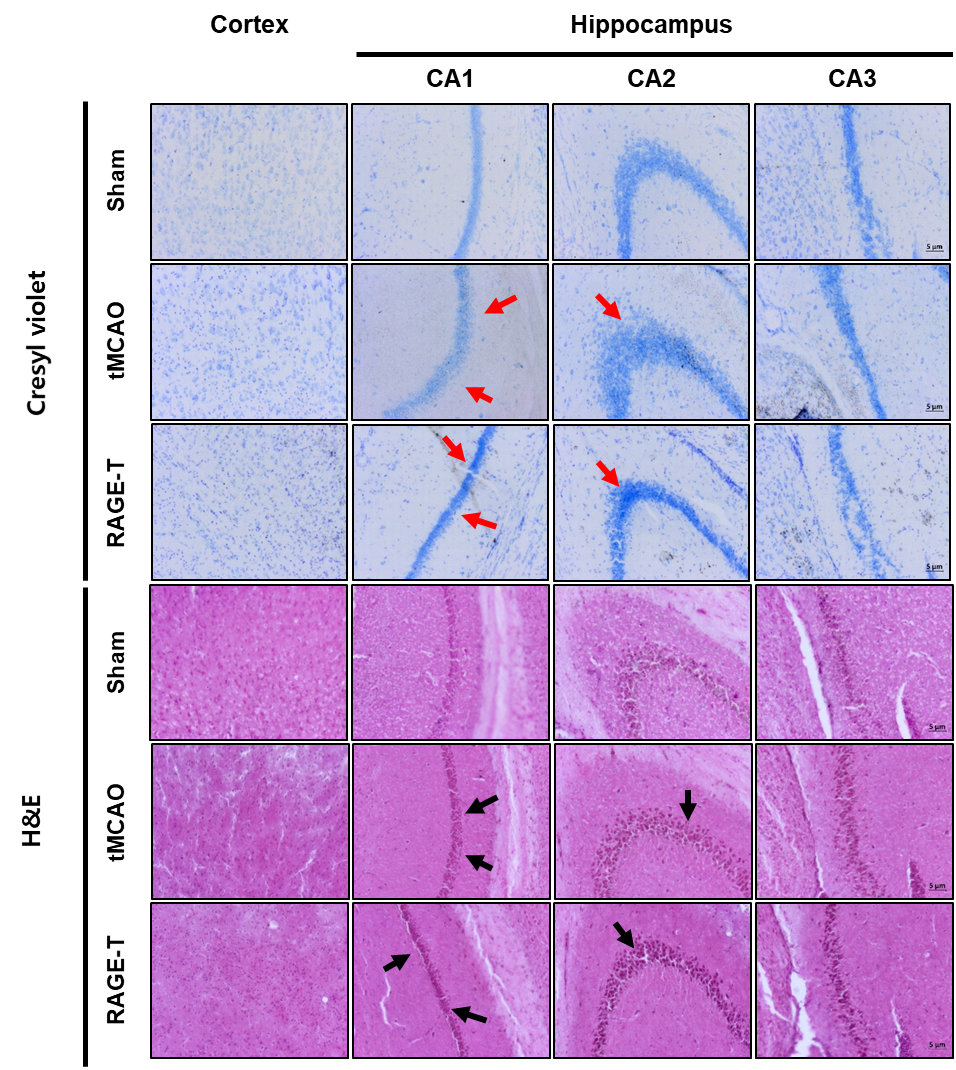


Fig. 1S Neuroprotective effects of RAGE on tMCAO-induced hippocampal (CA1, CA2, and CA3, respectively) cell death. Representative photomicrographs of cersyl violet staining and H&E staining of hippocampal regions corresponds with each other.


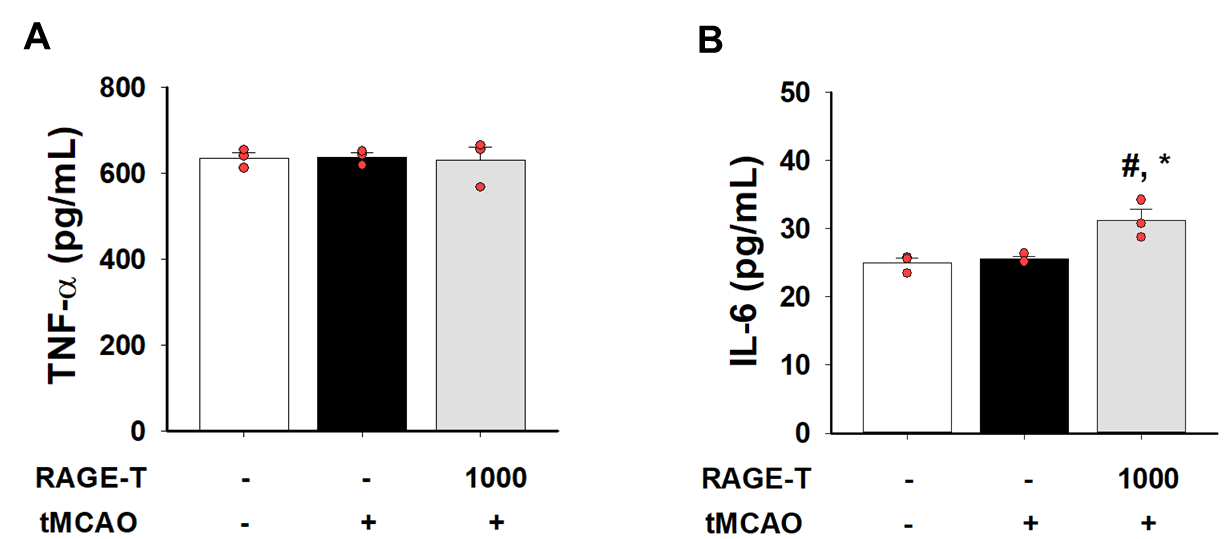


Fig. 2S Quantitative analysis of the TNF-α and IL-6 expressions in tMCAO-induced blood serum of mice. All data are expressed as mean ± SE (n = 3). # *p* < 0.05 vs. sham-operated group; * *p* < 0.05 vs. tMCAO group.
